# A randomized, double-blind, placebo-controlled multicenter clinical trial of Xiehuang Jiejing granule in the treatment of cough variant asthma in children

**DOI:** 10.1097/MD.0000000000031636

**Published:** 2022-11-18

**Authors:** Yi-Na Qiao, Shuang-Zhu Lin, Xiao-Zheng Duan, Ming-Hang Yang, Xiao-Fang Zhang, Jing-Jing Li, Sai-Nan Kang, Yu-Ting Wang, Ying Zhang, Xiao-Chun Feng

**Affiliations:** a Changchun University of Chinese Medicine, Changchun, China; b Department of Respiratory Medicine, Children’s Diagnosis and Treatment Center, Affiliated Hospital of Changchun University of Traditional Chinese Medicine, Changchun, China; c Center for Evidence-based Traditional Chinese Medicine, Beijing University of Chinese Medicine, Beijing, China.

**Keywords:** cough variant asthma, randomized controlled trial, study protocol, Xiehuangjiejing (XHJJ) granule

## Abstract

Cough variant asthma (CVA), also called concealed asthma or allergic asthma, is the most common cause of chronic cough in children. The disorder is mainly characterized by a nonproductive dry cough associated with a high recurrence rate that is conventionally treated with antibiotics, anti-inflammatory medications, cough suppressants, or expectorants. For millennia, Chinese herbal medicine (CHM) has been used widely in China to treat pediatric CVA cases, although high-quality evidence of CHM efficacy is lacking. In this study, the effectiveness and safety of Xiehuangjiejing (XHJJ) granule will be evaluated when used alone to treat children with CVA.

**Methods and analysis:**

A randomized, double-blind, parallel, placebo-controlled multicenter trial will be conducted over the course of 2 weeks. A total of 180 CVA patients of ages between 4 and 7 years old will be randomly assigned to the experimental group (XHJJ granules, 4.5 g administered 3 times daily) or control group (matched placebo, 4.5 g administered 3 times daily) in a 2:1 ratio based on subject number per group, respectively. The trial will consist of a 7-day medical interventional stage and a 7-day follow-up stage. On day 7 of the follow-up stage, an evaluation of all subjects will be carried out to assess cough symptom score as the primary outcome and several secondary outcomes, including TCM (traditional Chinese medicine) syndrome score, lung function, and dosage of salbutamol aerosol inhaler therapy. Safety assessments will also be evaluated during the trial.

**Discussion:**

The aim of this study was to examine the effectiveness and safety of Xiehuangjiejing (XHJJ) granule using a trial protocol designed to yield high-quality, statistically robust results for use in evaluating CHM as a treatment for CVA in children.

## 1. Introduction

Cough variant asthma (CVA) was first reported by Corrao in 1979^[1]^ as a disorder that is mainly associated with the clinical symptom of chronic cough without wheezing. This presentation differs from that of typical asthma in spite of the fact that CVA and asthma share pathogenic characteristics of persistent chronic airway inflammation and airway hyper-responsiveness. After CVA was first described in 1980 in 6- to 16-year-old children, results of subsequent studies demonstrated CVA was a common cause of chronic cough (without wheezing or dyspnea) in adults as well.^[2,3]^ Meanwhile, results of another study revealed that CVA accounted for 24% to 29% of chronic cough cases in Europe,^[4]^ while results of a multicenter study of 704 adult patients with chronic cough in 9 hospitals in 8 cities within 5 regions of China revealed CVA rates approaching 32.6%.^[5]^ With regard to pediatric CVA, results of an investigation of etiologies of chronic cough in Chinese children indicated that CVA was diagnosed in 41.95% (1900/4529) of cases, among which CVA rates were highest in children of 3 to 6 years of age.^[6]^ Notably, results of another study revealed that 1/3 of children aged 6 to 14 years with CVA who did not receive timely, effective treatment for the condition subsequently developed typical signs and symptoms of asthma.^[7]^

At present, inhaled corticosteroids (ICSs), oral corticosteroids, bronchodilators (β2-agonists), or leukotriene receptor antagonists are recommended treatments for CVA in adults and children,^[8]^ although ICS treatment efficacy is not known.^[9]^ On the one hand, studies^[10]^ have shown that as compared to oral corticosteroids, ICS administered using a nebulizer works faster and is accompanied by fewer systemic side effects and is thus more appropriate for patients, especially young children, with acute CVA. On the other hand, long-term administration of ICS therapy can cause a hoarse voice, pharyngeal discomfort, and candida infection that usually lead to poor clinical compliance^[11]^ that increased risk of disease relapse, results of one empirical study revealed that discontinuation of ICS for 6 months was associated with a cumulative CVA symptom relapse rate approaching 67%.

Chinese herbal medicine (CHM), which is currently widely used for the treatment of CVA in China,^[12,13]^ appears to be a valid treatment option for pediatric CVA cases, with results of a systematic review and meta-analysis^[14]^showing CHM to be effective and associated with fewer adverse effects relative to other CVA treatments. In fact, CHM administered with combination therapy consisting of ICS together with long-acting beta agonists (ICS/LABA) has been shown to be superior to ICS/LABA alone at reducing serum levels of inflammatory factors (e.g., IL-10, IL-6, TNF), reducing eosinophil levels,^[15]^ and inhibiting airway remodeling.^[16]^

Although many clinical studies have demonstrated that CHM treatment of CVA provides curative effects, many of those studies were methodologically limited due to unclear randomization methods, small sample sizes, inadequate follow-up measures, and so on. Consequently, reliable evidence showing the effectiveness of CHM as a treatment for CVA treatment is lacking, prompting this study to evaluate CHM for inclusion in clinical CVA treatment guidelines as an alternative treatment for the disorder.

Based on traditional Chinese medicine theory, a prescription known as Xiehuangjiejing (XHJJ) granule, which consists of ephedra (mahuang), scorpio (quan xie), Sophorae Flavescentis Radix (kusheng), Semen Armeniacae Amarum (xingren), Ginkgo biloba Linn (baiguo), and Radix Glycyrrhizae (gancao), can warm the lungs, dispel wind, relieve coughing, and reduce sputum production. In fact, most Chinese herbal ingredients in the formulation have been shown to exert beneficial health effects when used as asthma treatments. For example, ephedrine, a main active component of ephedra (mahuang), has been experimentally shown to induce anti-inflammatory and anti-allergic effects by inhibiting release of allergy-associated mediators and promoting selective shrinkage of nasal mucosal blood vessels.^[17,18]^ In recent years, explorations of medicinal asthma treatment formulations have intensified and become increasingly focused on ingredients obtained from insect species,^[19,20]^ such as earthworm (dilong), scorpion (quanxie), silkworm (jiangcang), and cicada (chantui). For example, scorpion venom polypeptides BmKbpp and BmKn2 have been shown to exert beneficial health effects, whereby BmKbpp has been reported to inhibit growth of gram-negative bacteria and BmKn2 has been reported to inhibit *Staphylococcus aureus* growth.^[21,22]^

Previous animal experiments and clinical observations have shown XHJJ prescription to be superior to other treatments in relieving cough, eliminating phlegm, improving lung function, and alleviating airway inflammation. In addition, XHJJ treatment of children with CVA was shown to significantly reduce total numbers of inflammatory cells, numbers of cells belonging to various inflammatory cell subpopulations, and levels of cytokines TNF, IL-6, and IL-1 in bronchoalveolar lavage fluid. Meanwhile, XHJJ treatment of asthmatic mice was shown to down-regulate expression of mitogen-activated protein kinases, the serine–threonine protein kinase Akt, vascular endothelial growth factor, and matrix metallopeptidase 9.^[23,24]^

## 2. Methods/design

### 2.1. Patient and public involvement

Patients or the public were not involved in the design, or conduct, or reporting, or dissemination plans of our research.

### 2.2. Study design

#### 2.2..1. Ethics, consent, and permissions.

This protocol was designed based on implementation of a randomized, parallel, placebo-controlled, multicenter trial. Participants, investigators, outcome assessors, statisticians, and others involved in conducting this trial will be blinded. The trial has been approved by the Institutional Ethics Committee of Jilin Provincial Hospital of Chinese Medicine CCZYFFLL2020-067 and is registered in the Chinese Clinical Trial Registry (ChiCTR2000040906). The study will be conducted in accordance with the principles outlined in the Declaration of Helsinki (2013 version). SPIRIT (Standard Protocol Items: Recommendations for Intervention Trials) guidelines^[25]^ (Additional file 1) will be followed while reporting the protocol for the trial, while CONSORT (Consolidated Standards of Reporting Trials) will be followed while reporting the final results. Written informed consent with regard to the purpose, possible risks, and benefits of this trial will be obtained from each participant prior to enrollment in the trial.

#### 2.2..2. Study design and procedure.

**Figure 1.**
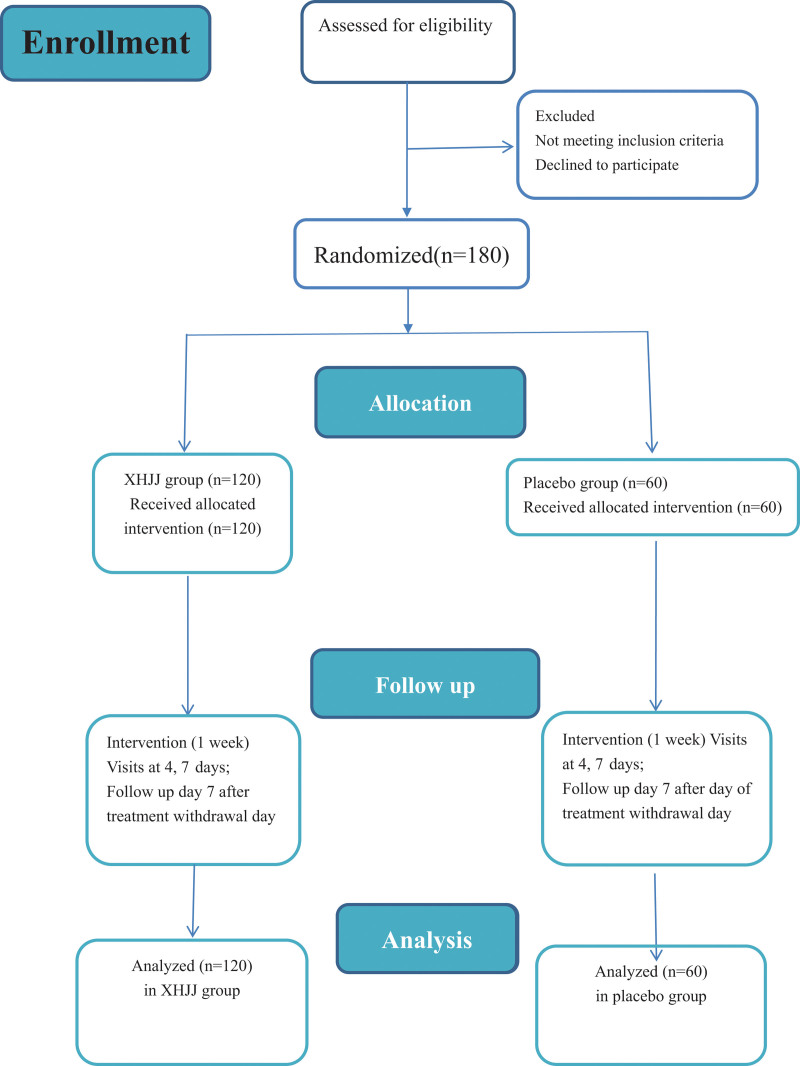
Flowchart of this study.

This trial will consists of a 7-day medical intervention stage and a 7-day follow-up stage (Figure 1)and will be carried out concurrently in 6 hospitals in China: the Affiliated Hospital of Changchun University of traditional Chinese medicine (TCM), Changchun; the Affiliated Hospital of Beijing University of TCM, Beijing; the Affiliated Hospital of Liaoning University of TCM, Shenyang; the Affiliated Hospital of Shandong University of TCM, Shandong; the Jiangsu Hospital of TCM, Nanjing; and the First Affiliated Hospital of Henan University of TCM, Henan.

Patients will be invited to participate in an initial assessment (first visit), during which they will be selected or rejected for enrollment according to results of screening based on inclusion and exclusion criteria. Next, eligible patients will receive physical examinations to assess TCM syndrome scores, as well as laboratory testing that will include whole blood counts, routine blood and urine testing, liver and kidney function testing, electrocardiography, and chest radiography. Participants will be randomly assigned to the experimental group (receiving XHJJ granule) or the placebo group and will be blinded with regard to group assignments. Patient self-reported cough symptom scores and TCM syndrome scores will be recorded daily in patient diaries during the treatment period.

Patient face-to-face interviews with blinded assessors will be scheduled on the fourth day (second visit) in order to collect relevant clinical data, including lung function assessments and blood tests, as well as data pertaining to quality of life, safety, and drug usage. Beginning on the seventh day (third visit) and throughout the second week (the follow-up stage), participants will continue to self-report cough symptom scores and TCM syndrome scores daily in their diaries and document their utilization of health resources (including any drug use). During the last visit on day 7, quality of life, lung function, adverse events (AEs), and drug usage will be assessed and participants will undergo blood tests. An overview of specific measurements and time points for data collection is presented in (Table 1).

**Table 1:** Measurement items and point of data capture.

Study period	Screening period (d)	Medication (d)		Safety follow-up
	Visit 1 (−7 to 0)	Visit 2 (4 d)	Visit 3 (7 d)	
Sign informed consent	√			
Fill in general information	√			
Medical history and treatment history	√			
Physical examination	√		√	
chest radiographs	√			
Lung function	√		√	
TCM syndrome score	√	√	√	
Vital signs	√	√	√	√
Routine blood test	√		√	√
Routine urine test	√		√	√
ECG, liver, and kidney function	√		√	√
Adverse event		√	√	√
Distribution of experimental drugs	√	√		
Test drug recovery		√	√	
Issue diary card	√			
Recycle patient’ s diary card			√	
Comorbidity and medication records	√	√	√	
Compliance evaluation		√	√	
Safety assessment		√	√	

### 2.3. Participants

Participants will be recruited based on hospital pediatric outpatient departmental records and invitations to join the study as advertised using posters, newspapers, and online ads. Participants will be included only if they meet all of the following inclusion criteria: meet diagnostic criteria for CVA^[7]^ and a diagnosis of wind and cold attacking the lung^[26]^ are within the age range of 4 to 7 years, Cough symptom severity (daytime + nighttime) score ≧ 4, and Subjects and their guardians voluntarily participate in this clinical trial and provided a signed consent form.

### 2.4. TCM syndrome pattern differentiation

The Chinese syndrome pattern differentiation type indicating pattern of wind and cold attacking the lung will be based on guidelines delineated in the Clinical Research of New Investigational Drugs in Traditional Chinese Medicine, and Guidelines for the Prevention and Treatment of pediatric CVA in Traditional Chinese Medicine.^[27]^ The diagnostic standards are as follows:

1. Primary signs and symptoms is cough and evident at night or in the morning.
2. Secondary signs and symptoms include little or no phlegm; pharyngeal itching; flow cleaning stuff. The hypoglossal veins are pale purple; turbinate light red and slightly swollen.

Participants will be diagnosed with deficiency of wind and cold attacking the lung syndrome if they have the primary signs or symptoms, at least 3 of the secondary signs or symptoms, and examination of the tongue and pulse indicates syndrome of wind and cold. Investigators at each site will administer a symptom assessment survey to every participant. This will ensure standardized scoring across sites. After being screened for inclusion and exclusion criteria, eligible patients will be entered into the trial.

### 2.5. Exclusion criteria

Patients with one or more of the following characteristics will be excluded: chronic cough due to non-CVA causes; previous receipt of treatments with glucocorticoids, leukotriene modulators, long-acting β receptor agonists, and/or theophylline within 2 weeks prior to enrollment; signs of allergic reactions to the test drug or some components of the drug; Combined with other serious diseases of the system; a history of participation in other trials within the 3 months prior to screening for this trial; and affliction with other conditions that lead the investigator to reject the patient as a participant in this clinical trial.

### 2.6. Interventions

#### 2.6..1. Experimental group.

XHJJ (herb granule, 4.5 g per packet) will be obtained from Hefei Huarun Sanjiu Pharmaceutical Co., Ltd. Participants in the experimental group will be instructed to orally consume one packet of XHJJ dissolved in warm water 3 times per day for 7 days, with each dose consumed one-half hour after a meal.

#### 2.6..2. Control group.

A placebo treatment to simulate XHJJ prescription granules will be produced and packaged by the same manufacturer so that the placebo and XHJJ treatments cannot be distinguished from one another based on appearance of granules and packaging, solubility in warm water, taste, smell, dosage, and method of administration.

Only qualified personnel in each site may dispense drugs to participants and they will make sure that the medicine is kept sealed in a cool, dry, and secure locked place. Drug packaged in each site on returned of the unfinished/empty at every visit, relevant counts and return dates will be recorded on case report forms (CRFs).

### 2.7. Concomitant medications

For severe cough symptoms (cough score ≥ 3), patients will be permitted to temporarily use salbutamol aerosol (Ventolin) as long as they accurately record numbers and times of administration of spray doses. Use of other TCMs, glucocorticoids, and beta agonists, etc. will be prohibited in order to prevent introduction of potential biases into results of the trial.

### 2.8. Randomization, concealment, and blinding

All eligible patients will be randomly assigned to either the experimental group or the control group in a 2:1 ratio reflecting number of subjects per group, respectively. The block randomization sequence will be generated using SAS 9.2 software (SAS, Cary, NC) by an independent statistician, who will not be involved in data analysis.

The allocation sequence will remain confidential prior to unblinding to ensure that all patients, investigators, outcome assessors, and statisticians involved in data analyses will not learn the allocation schedule during the trial. If a major medical emergency occurs, for example serious AEs for which knowledge of the allocation schedule might influence clinical treatment, the corresponding subject’ s randomization code may be obtainable by a requestor who submits an emergency letter to an authorized principle investigator representing the study site associated with the AE.

### 2.9. Outcome measures

#### 2.9..1. Primary outcome.

The primary outcome will be based on the cough symptom score.^[28]^ Mean scores of daytime or nighttime cough severity will be calculated as equal to the total score of daytime or nighttime cough severity divided by the total number of investigational days, respectively.

#### 2.9..2. Secondary outcomes.

1. TCM syndrome score;
2. Lung function;
3. Dosage of salbutamol aerosol.

#### 2.9..3. Other secondary outcomes.

Laboratory examination findings will be derived from chest X-rays and results of serum allergen-specific IgE detection tests and nitric oxide detection tests. Overall health resource utilization data will be recorded, including doctor visits, hospitalizations, and medication usage.

### 2.10. Safety assessments

The following safety assessments will be performed as follows:

Vital signs: blood pressure, respiration, body temperature, heart rate, etc. at baseline, on day 4, and on day 7; Blood and urine samples, liver function, renal function, electrocardiograms, and chest X-rays will be obtained at the end of the run-in period and at day 4 and day 7. Researchers will be alerted to focus on changes in results occurring among the different time points.

AEs: an AE is any adverse medical event that occurs during the trial, regardless of whether it is related to the drug under evaluation. Patients will be required to report all AEs at each visit. AE characteristics, degree of severity, time of occurrence, duration, AE treatment protocols, and follow-up treatments of AEs will be recorded on case report forms. All AEs will be reviewed to assess causal relationships between AEs and investigational drug according to the standards developed by the Uppsala Monitoring Centre of the World Health Organization.

### 2.11. Termination and withdrawal

Participants may withdraw from the trial for any reason at any time. Withdrawal or dropped visits will also be recorded on CRFs. Investigators will review the CRFs while considering inclusion, exclusion, and withdrawal criteria. Reasons for withdrawal or termination and the last medication time will be detailed in CRFs. All withdrawn and terminated cases will be reported and analyzed.

### 2.12. Sample size estimation

Interpretation of findings by clinical experts. Minimum sample size will be based on a total of 160 subjects (106 vs. 53) that will be assigned to experimental and control groups in a ratio of 2:1 (based on subject number per group). In order to detect any differences between groups with a power of at least 80%, the type I error rate will be calculated based on a 2-sided error detection cutoff of 0.05. If enough subjects can be recruited, the target sample size will be adjusted to include 180 subjects to compensate for loss of subjects as based on a predicted dropout rate of 20%. The 180 subjects will be allocated to groups based on the above mentioned 2:1 ratio (120 subjects in the CHM group, 60 subjects in the placebo group).

### 2.13. Data management and quality control

All investigators on the study team will be trained to follow standard operating procedures and strictly adhere to the protocol during the entire study period. Investigators will be reminded to finish CRFs completely and correctly in a timely manner according to information obtained from original medical records. Lung function and blood tests will be conducted by qualified medical professionals.

Investigators from the Affiliated Hospital of Changchun University of TCM will visit each site once a month to audit the quality of data collection and resolve any issues encountered at the sites. All documents will be properly secured and preserved under confidential conditions and archived.

### 2.14. Statistical analyses

Statistical analysis will be conducted by an independent statistician from the Beijing University of Chinese Medicine. The statistician will follow a detailed statistical analysis plan that was drafted separately by a different statistician before trial inception. Results of statistical analysis will be finalized before trial unblinding. Baseline characteristics will be described using mean ± SD for continuous data and number and percentage for categorical and hierarchical data. Inferential analysis of continuous data will rely on use of *t* tests or the Wilcoxon rank sum test depending on whether the assumption of normal distribution is met or not. Chi-squared or Fisher’s exact tests will be used to compare categorical data between groups. ANCOVA will be applied when the assumption is met for analysis of the primary outcome, with the covariates including baseline score and study site. In addition, the interaction between study site and group will be considered in this model.

The main analyses to determine drug effectiveness will follow intention to-treat (ITT) analytical principles, with the definition of ITT agreed upon and finalized before trial unblinding. Analysis of the per-protocol set of data will be conducted and the results will be used to complement results of ITT analysis. The safety analysis set of data will be the main data set used for safety analyses.

## 3. Discussion

To date, the overall quality of randomized controlled trials conducted to evaluate TCM therapies has not generally been high,^[29]^ due to poor study design and methodological limitations. While designing the current pioneering investigation to evaluate the effectiveness of CHM treatment alone for pediatric CVA, we took steps to guarantee the quality of the study by ensuring that conclusions will be verified and that trial design and study implementation will strictly adhere to quality control protocols. For instance, a training session will be held at each site to explain the study protocol and how TCM syndrome pattern differentiation should be performed in order to maximize consistency of results obtained by different clinicians. Additionally, biochemical testing will be performed by the independent laboratory associated with each hospital to ensure that reliable high-quality data are generated. We hypothesize that children with CVA will benefit from treatment with XHJJ granule. In order to minimize effects of selection bias and observer bias on results, strict adherence to trial protocols designed to minimize such biases will be maintained. Together, these measures should ensure that results obtained from this trial will reveal the efficacy of XHJJ granule as an alternative therapy to treat children with CVA.

## Author contributions

**Conceptualization:** Shuang-zhu Lin.

**Data curation:** Ying Zhang, Yi-na Qiao.

**Funding acquisition:** Xiao-chun Feng.

**Methodology:** Ying Zhang, Xiao-zheng Duan.

**Project administration:** Ming-hang Yang, Xiao-fang Zhang.

**Investigation:** Jing-jing Li, Sai-nan Kang, Yu-ting Wang.

**Writing—original draft:** Yi-na Qiao.

**Writing—review and editing:** Xiao-chun Feng, Shuang-zhu Lin.

## References

[1] Niimi A Matsumoto H Mishima M. Eosinophilic airway disorders associated with chronic cough. Pulm Pharmacol Ther. 2009;22:114–20. 19121405 10.1016/j.pupt.2008.12.001

[2] Tajiri T Niimi A Matsumoto H. Prevalence and clinical relevance of allergic rhinitis in patients with classic asthma and cough variant asthma. Respiration. 2014;87:211–8. 24401902 10.1159/000355706

[3] Cao Y Lin SH Zhu D. WeChat public account use improves clinical control of cough-variant asthma: a randomized controlled trial. Med Sci Monit. 2018;24:1524–32. 29536984 10.12659/MSM.907284 PMC5865451

[4] Desai D Brightling C. Cough due to asthma, cough-variant asthma and non-asthmatic eosinophilic bronchitis. Otolaryngol Clin North Am. 2010;43:123–30, x. 20172262 10.1016/j.otc.2009.11.006

[5] Lai K Chen R Lin J. A prospective, multicenter survey on causes of chronic cough in China (Chinese). Chest. 2013;143:613–20. 23238526 10.1378/chest.12-0441

[6] Lai KC Lin J. Chinese children’s chronic cough etiology composition multicenter study (Chinese). Chin J Pediatr. 2012:83–92.

[7] Xue B Stephen OA Wang H. Application software for diagnosis and management of cough executive summary: ACCP evidence-based clinical practice guidelines (Chinese). Chin Med Assoc Resp Ann Meeting. 2013:748.

[8] Tagaya E Kondo M Kirishi S. Effects of regular treatment with combination of salmeterol/fluticasone propionate and salmeterol alone in cough variant asthma. J Asthma. 2015;52:512–8. 25329681 10.3109/02770903.2014.975358

[9] Morice AH Millqvist E Bieksiene K. ERS guidelines on the diagnosis and treatment of chronic cough in adults and children. Eur Respir J. 2020;55:1–115. 10.1183/13993003.01136-2019 PMC6942543 31515408

[10] Szefler SJ Eigen H. Budesonide inhalation suspension: a nebulized corticosteroid for persistent asthma. J Allergy Clin Immunol. 2002;109:730–42. 11941331 10.1067/mai.2002.122712

[11] Lin JT Wang WQ Zhou X. Trends of asthma control, disease management and perception in China (Chinese). Zhonghua Jie He He Hu Xi Za Zhi. 2018;41:191–5. 29518847 10.3760/cma.j.issn.1001-0939.2018.03.009

[12] Fan RR Wen ZH Wang DW. Chinese herbal medicine for the treatment of cough variant asthma: a study protocol for a double-blind randomized controlled trial. Trials. 2019;20:3. 30606237 10.1186/s13063-018-3073-x PMC6318874

[13] Zhang ZD Deng YQ Zhang Y. TCM differential treatment of cough variant asthma. J Tradit Chin Med. 2010;30:60–3. 20397466 10.1016/s0254-6272(10)60015-5

[14] Song P Zeng L Liang Z. Clinical efficacy and safety of Chinese herbal medicine auxiliary therapy for childhood cough variant asthma: a systematic review and meta-analysis of 20 randomized controlled trials. Intern Med. 2016;55:2135–43. 27522988 10.2169/internalmedicine.55.5546

[15] Zhou QL Wang L. Effect of Shu Feng Xuan Fei Decoction on airway inflammatory factors in patients with cough variant asthma. Chin Herbal Med. 2017;40:2971–3.

[16] Lu FW Chen ZB. Majing Zhike Granule in treating 37 patients with cough variant asthma of wind exuberance syndrome (Chinese). Fujian Trad Chin Med. 2017;48:1–3.

[17] Vansal SS Feller DR. Direct effects of ephedrine isomers on human beta-adrenergic receptor subtypes. Biochem Pharmacol. 1999;58:807–10. 10449190 10.1016/s0006-2952(99)00152-5

[18] Hong H Chen HB Yang DH. Comparison of contents of five ephedrine alkaloids in three official origins of Ephedra Herb in China by high-performance liquid chromatography. J Nat Med. 2011;65:623–8. 21465337 10.1007/s11418-011-0528-8

[19] Wu LF Xu RQ. Scorpion powder, treatment of 30 children with cough variant asthma clinical observation (Chinese). Sichuan Trad Chin Med. 2007:665–7.

[20] Yi JZ. Treated 32 cases of cough variant asthma with syndrome differentiation (Chinese). Jiangxi Trad Chin Med. 2000:21.

[21] Wang Z Wang W Shao Z. Eukaryotic expression and purification of anti-epilepsy peptide of Buthus martensii Karsch and its protein interactions. Mol Cell Biochem. 2009;330:97–104. 19370317 10.1007/s11010-009-0104-7

[22] Wang XG Zhu DD Li N. Scorpion venom heat-resistant peptide is neuroprotective against cerebral ischemia-reperfusion injury in association with the NMDA-MAPK pathway. Neurosci Bull. 2020;36:243–53. 31502213 10.1007/s12264-019-00425-1 PMC7056763

[23] Zheng MZ. Granules XieHuang on asthma mice p38 lightning MAPK and Th1/Th2cytokines changes (Chinese). Changchun Univ Chin Med. 2014;03.

[24] Hong TY. Granules XieHuang affect the regulatory mechanism of NF-κB and HIF-1α signaling pathway in the treatment of asthma (Chinese). Changchun Univ Chin Med. 2020;01.

[25] Chan AW Tetzlaff JM Gøtzsche PC. SPIRIT 2013 explanation and elaboration: guidance for protocols of clinical trials. BMJ. 2013;346:e7586. 23303884 10.1136/bmj.e7586 PMC3541470

[26] Feng XD Sun L. Chinese medicine pediatric clinical diagnosis and treatment guidelines · pediatric cough variant asthma (developed) (Chinese). J Pediatr Trad Chin Med. 2016;12:1–4.

[27] Zhu WF. Description of the formulation of the diagnostic criteria for common witnesses of traditional Chinese Medicine (Trial implementation) (Chinese). 9th TCM Diag Acad Conf China Assoc Trad Chin Med. 2008:15–9.

[28] Asthma Group, Respiratory Society of Chinese Medical Association. Guidelines for diagnosis and Treatment of Cough (2009 edition) (Chinese). Chin J Gen Practit. 2009;09:608–13.

[29] He J Du L Liu G. Quality assessment of reporting of randomization, allocation concealment, and blinding in traditional Chinese medicine RCTs: a review of 3159 RCTs identified from 260 systematic reviews. Trials. 2011;12:122. 21569452 10.1186/1745-6215-12-122 PMC3114769

